# Cardiorespiratory Fitness in Children: A Simple Screening Test for Population Studies

**DOI:** 10.1007/s00246-014-0960-0

**Published:** 2014-07-29

**Authors:** Marek Jankowski, Aleksandra Niedzielska, Michał Brzezinski, Józef Drabik

**Affiliations:** 1 Center for Promotion of Children’s Health and Fitness, ul. Kołobrzeska 61, 80-397 Gdansk, Poland; 2 Department of Pediatrics, Gastroenterology, Hepatology, and Children Nutrition, Medical University of Gdansk, ul. Nowe Ogrody 1-6, 80-803 Gdansk, Poland; 3 University of Bydgoszcz, ul. Unii Lubelskiej 4C, 85-059 Bydgoszcz, Poland

**Keywords:** Cardiorespiratory fitness, Heart rate, Reference range, Step test, Screening

## Abstract

Cardiorespiratory fitness is one measure of body functions, and its assessment should play an important role in the activities associated with the promotion of physical activity as an important component of a healthy lifestyle. This study aimed to develop a reference system of the mean post-exercise heart rate (HR_mean post-ex_) after a 3-min step test for use in screening the cardiorespiratory fitness of 6- to 12-year-old children. The study included 14,501 children ages 6–12 years from primary schools in Gdansk. The participants were subjected to the 3-min Kasch Pulse Recovery Test (KPR Test). The reference range for the classification of cardiorespiratory fitness was developed on the basis of the age-specific percentile distribution of HR_mean post-ex_ in 6- to 9- and 10- to 12-year-old children. This study showed that the 3-min KPR Test is easy to perform and well tolerated by school-age children. As such, it can constitute a useful tool for health promoters and educators. The presented age- and gender-specific reference range of HR_mean post-ex_ enables the assessment and monitoring of submaximal exercise-induced changes in the cardiovascular system and, consequently, the physical fitness of a given individual.

Physical fitness, including cardiorespiratory fitness, is an expression of the health potential of a human being. In addition to other parameters, physical fitness is characterized by the post-exercise heart rate (HR), considered to be an indicator of cardiorespiratory fitness [5, 10]. Post-exercise restitution capacity and exercise HR were identified in a factorial analysis as belonging to the group of energy capacities with an aerobic basis [20].

Individuals with high values of peak oxygen uptake (VO_2 max_) are characterized by the ability to restore all pre-exercise reactions rapidly and a low HR during submaximal exercise [22]. Consequently, the value of post-exercise HR obtained with the step test is considered to be an indicator of cardiorespiratory fitness [9, 10]. Therefore, the response of the circulatory system during and after submaximal exercise fits within the expanded classical definition of physical fitness [21] and is closely associated with endurance. In this context, the aforementioned step test also can be considered an endurance trial in the narrow sense. Unambiguously, however, it represents the circulatory system’s attempt to respond to submaximal exercise.

Evaluation of cardiorespiratory fitness requires easy, reproducible screening tests that can be conducted under the conditions of an epidemiologic study. Currently, a small number of screening methods enable simple determination of cardiorespiratory fitness, particularly in children. Most of these methods need a special environment and special conditions [15]. Such examination can provide parents, teachers, and physicians with valuable information concerning the lifestyle and general health status of the child, as well as with long-term data on the risk factors of civilization-related disorders.

One of the most typical places in which a simple, quick screening test can be used is the school. Easy to perform during physical education classes, it can give reliable information about health status, changes in physical activity, and cardiac fitness during the school year. It also can be used as a simple diagnostic tool to divide children into groups according to their physical potential for needing physical education classes. This can be a way of preventing negative behaviors in the group of better prepared pupils and can give us information about needs in training of children with worse cardiorespiratory fitness.

Cardiorespiratory fitness is one measure of body functions, and its assessment should play an important role in the activities associated with the promotion of physical activity as an important component of a healthy lifestyle. This study aimed to develop a reference system of mean post-exercise HR (HR_mean post-ex_) after a 3-min step test used to screen the cardiorespiratory fitness of 6- to 12-year-old children.

## Methods

All the procedures in this study were approved by the Local Ethics Committee of the Medical University in Gdansk. The study included 14,501 children ages 6–12 years from primary schools in Gdansk. The study excluded 219 children due to a high HR during the test. The age of the children was 6.00–12.99 years, calculated as the difference between the date of the test and the date of birth. The age was calculated with precision to 0.01 year (e.g., children 6.00–6.49 years of age were classified in the 6-years-of-age category), as suggested in the literature [17]. The results achieved by overweight and obese children were included to determine the gender- and age-specific reference system of HR_mean post-ex_ for the whole population.

Standard body mass index (BMI) values were used to assess body mass percentiles [6]. The number of overweight and obese individuals was 3,210 (1,624 boys and 1,586 girls), which corresponded to 22.13 % of all the examined children (22.66 % of the boys and 21.61 % of the girls). Because obesity and overweight represent established factors influencing cardiorespiratory fitness in children [23], obese and overweight children also were included in the study.

Children 6–7 years of age were assessed between January 2008 and June 2010, whereas 8- to 12-year-old children were examined between January 2007 and December 2009. The testing of the 6- to 7-year-old subjects took place at the Center for the Promotion of Kids’ Health and Fitness within the framework Your Child’s Healthy Life health program. The 8- to 12-year-old children were examined at their educational centers within the framework of the Healthy Student program.

The study excluded only children with comorbidities or absolute contraindications to physical exercise due to cardiovascular strain based on a parent or caregiver interview, physical examination, and data from medical records. For each participant, the decision to qualify for the exercise test was made by the physician.

The analyzed variables included the measurements of body height and weight, as well as HR_mean post-ex_. Anthropometric measurements were taken during morning hours, with the children barefoot and dressed in their gym outfits. The measurements were taken with the children the standing position using the Mensor WE150 (Mensor AJ, Warsaw, MAZ, Poland) scale with a height meter. Body height was measured to the nearest 0.001 m and body weight to the nearest 0.1 kg. The scale was calibrated every day.

The participants were subjected to a 3-min Kasch Pulse Recovery Test (KPR Test) [5, 12]. The KPR Test consisted of climbing a 0.305-m step at a rate of 24 steps up and down per minute. The rate of climbing was defined by a metronome set at 96 beats (signals) per minute. The HR was recorded with the “Polar” electronic analyzer (Polar T31, Polar Electro, Kempele, Finland). Throughout the test, HR was monitored continuously (i.e., during 3 min of exercise [step test] and during 1 min and 5 s of recovery [seated position]). Only the values of post-exercise HR (i.e., the values recorded 1 min after completion of the test [no later than 5 s]) were included in the further analysis. All HR characteristics were recorded during restitution with the participant in the seated position (subjects were instructed to sit still, breath normally, and not engage in conversation). An arithmetic mean value calculated from these results (HR_mean post-ex_) was the principal variable used in further analyses.

The test was considered incomplete whenever its protocol was not followed (e.g., due to an improper climbing rate, conversation, or refusal to exercise). The test was discontinued if the exercise HR exceeded 180 beats per minute for more than 15 s. A high HR during submaximal exercise corresponds to poorer cardiorespiratory fitness [22]. Consequently, continuation of the test in the case of a high-exercise HR was not necessary in the context of the cardiorespiratory fitness assessment. Moreover, continuation of exercise was associated with the risk of a further increase in HR to the expected level of HR_max_ in children (HR_max_ = 210 − [0.65 × age in years]) ± 10 % [11]. Consequently, motivating the participant to continue the exercise until refusal would correspond to a maximal test. In view of the potential health risks associated with exercise tests, particularly for less active individuals, discontinuation of the KPR Test in such cases was substantiated by safety reasons [13].

In 1970, the test used in this study was adopted by the YMCA as an “excellent cardiorespiratory test” [10]. Furthermore, it was used in epidemiologic studies [3, 19] and in health training programs [18].

The KPR Test belongs to a group of step tests used to estimate exercise capacity on the basis of HR_mean post-ex_ values. In all previously mentioned studies, the pulse of the participants was determined by palpation of the carpal or carotid artery, and all previously defined reference ranges referred to such a method of HR monitoring.

For practical reasons, our study used a modified method of post-exercise HR registration to facilitate its measurement and to reduce the risk of errors. We used an electronic device in the monitoring of HR. This necessitated the development of a new reference range corresponding to the modified method of HR measurement.

Two age brackets (ages of 6–9 and 10–12 years) corresponding to progressive growth and development were defined during the development of the reference range [14]. The limits of those age brackets were set at the age of 10 years, corresponding to the end of the juvenile–prepubertal period and the beginning of the juvenile period (puberty and growth). The reference range for the classification of cardiorespiratory fitness was developed on the basis of an age-specific percentile distribution of HR_mean post-ex_ in children 6–9 and 10–12 years of age.

The values of HR_mean post-ex_ used in the reference range were determined based on gender-specific arithmetic means of HR_mean post-ex_ calculated for each 0.5-year age bracket (e.g., the 50th percentile of HR_mean post-ex_ in 6- to 9-year-old boys corresponded to the arithmetic mean of the 50th percentiles for 6-, 7-, 8-, and 9-year-old boys). The reason for using such a method of determination was to eliminate the mathematical influences on the final value of quantitative differences between particular age cohorts.

### Statistical Analysis

All statistical analyses were conducted with the Statistica PL package (StatSoft Polska Sp. z o.o., Krakow, MAL, Poland). The values of the given percentiles (5th, 10th, 15th, 25th, 50th, 75th, 85th, 90th, and 95th) were calculated for each 0.5-year age bracket separately for the boys and the girls. Percentiles enable an easy practical assessment of the scale and are easier for both parents and children to understand.

## Results

This study resulted in tables that are simple to use (Tables 1 and 2). They allow each interested person (teacher, physician, school nurse) to easily assess results of performed test for children ages 6–13 years.

**Table 1:** Percentile distribution of step test post-exercise heart rates of 6- to 12-year-old boys

Age (years)	*n*	Percentile								
		5th	10th	15th	25th	50^th^	75th	85th	90th	95th
6.0	958	95	97	100	104	111	120	125	129	137
6.5	1,386	94	97	99	103	110	120	125	128	135
7.0	552	92	96	97	102	109	119	124	128	133
7.5	65	96	99	102	107	117	132	139	140	145
8.0	286	97	100	104	107	118	128	135	138	145
8.5	392	97	101	104	108	118	129	136	141	148
9.0	428	95	100	103	108	118	130	137	142	148
9.5	412	96	98	103	108	118	129	136	140	148
10.0	384	92	96	100	104	116	127	135	139	147
10.5	407	91	96	97	104	116	128	135	140	147
11.0	407	92	95	98	103	114	126	134	138	146
11.5	396	92	97	100	104	117	129	135	140	148
12.0	419	91	95	97	104	116	130	137	142	150
12.5	358	92	97	98	104	115	129	138	143	149
Total	7,164	93	97	100	104	113	124	131	136	144

**Table 2:** Percentile distribution of step test post-exercise heart rates of 6- to 12-year-old girls

Age (years)	*n*	Percentile								
		5th	10th	15th	25th	50th	75th	85th	90th	95th
6.0	980	99	103	105	110	118	127	134	137	143
6.5	1,370	100	103	104	109	117	127	134	139	145
7.0	552	98	101	104	108	116	126	132	137	145
7.5	85	102	105	109	116	127	138	143	150	158
8.0	312	102	107	110	116	127	139	146	150	154
8.5	400	101	106	110	116	126	137	145	149	156
9.0	440	101	107	109	114	125	138	144	149	154
9.5	404	100	103	107	114	127	141	148	154	160
10.0	417	103	104	110	115	127	137	142	147	153
10.5	425	101	104	108	114	126	139	145	149	156
11.0	446	103	107	111	116	129	140	145	150	155
11.5	406	101	106	111	118	129	141	148	152	157
12.0	448	103	108	111	116	129	142	150	155	160
12.5	346	103	106	110	116	129	144	150	154	161
Total	7,337	100	104	107	112	122	135	142	147	154

The sample size of all the studied age groups and the percentile distributions of gender- and age-specific post-exercise HRs for the 5th, 10th, 15th, 25th, 50th, 75th, 85th, 90th, and 95th percentiles of all the examined children are presented in Tables 1 and 2. These tables show detailed percentile information that can be used during cardiorespiratory fitness testing of school children, sport testing, and drawing changes in personal results during sports training. These tables give physicians, teachers, and coaches easy-to-use, sex-specific information on individual cardiac efficiency.

Additionally, we present a shorter version of the charts that can be used during screening testing in nurses’ offices or the offices of general practitioners. The reference ranges of the modified KPR Test for the post-exercise HR of 6- to 9- and 10- to 12-year-old boys and girls are presented in Table 3.

**Table 3:** Classification for ranges of reference values of mean post-exercise heart rates of 6- to 12-year-old children

Cardiorespiratory fitness	Boys (6–9 years)	Boys (10–12 years)	Girls (6–9 years)	Girls (10–12 years)
Excellent (HR_mean post-ex_ < 5th %tile)	<95	<93	<100	<102
Very good (HR_mean post-ex_ ≤ 25th %tile)	95–106	93–105	100–113	102–116
Good (HR_mean post-ex_ ≤ 50th %tile)	107–115	106–116	114–123	117–128
Sufficient (HR_mean post-ex_ ≤ 75th %tile)	116–126	117–128	124–134	129–141
Poor (HR_mean post-ex_ ≤ 95th %tile)	127–142	129–147	135–152	142–157
Very poor (HR_mean post-ex_ > 95th %tile)	>142	>147	>152	>157

Both the full and short versions of the post-exercise HR distribution charts show the differences in the post-exercise HR of girls and boys in all age groups and percentiles. The causes for this need further investigation because there were no differences in pre-exercise HR. Although pre-exercise HR cannot be used as a diagnostic tool for children and adolescents due to its rapid changes in stressful situations (e.g., performing the test), the authors have seen statistically significant differences in the post-exercise HR of overweight and non-overweight children. These results were not included in this report because we wanted to present references for the whole population of children ages 6–12 years. Additionally the cause and result of a higher HR in overweight and obese children needs further investigation. This will be a subject of a different article.

## Discussion

According to the evidence published in physiology textbooks [21], VO_2 max_ determined by a direct method is an optimal determinant of physical fitness. However, determination of this parameter often is not possible, reliable, safe, or even necessary in otherwise healthy preadolescent children [24]. Reaching the maximal level of exercise can be impossible for some physically inactive children who do not practice sports and are not motivated to perform this test [8]. Consequently, in the course of epidemiologic studies, substitutes for this test are frequently used such as the time required for a treadmill run or the HR recorded during and after submaximal exercise [2, 4].

A number of authors have suggested that VO_2 max_ also can be estimated using step tests [1, 12], which can differ with regard to the climbing rate, the step’s height, test duration, number of steps, and the methods used to determine exercise capacity. The KPR Test used in this study belongs to such a group of step tests.

The authors of this study were guided by the normal level of physical fitness as the desirable target in determining the reference values of exercise capacity, taking into account future application of these reference limits in the promotion of physical activity. Using this tool to build reference range for the development of functional characteristics, particularly cardiorespiratory fitness, can be a substantiated instrument in health promotion. It should be remembered that in addition to being informed about the percentile score, the examined subject should be aware whether his or her fitness level is desirable in the context of health status [7]. Especially when using this test as a marker of health status, children and their parents as well as school teachers and managers should be given simple information about the results (i.e., positive or negative changes in physical activity status).

An important aspect of the testing tool in the educational context is discontinuation of approximately 1.5 % of tests due to an extremely high (>180 beats/min) exercise HR (for safety reasons). The authors recommend using the additional classification of “very poor exercise capacity–test discontinued” in such cases.

The authors also recommend the exclusion of children with obesity and overweight from the reference range of HR_mean post-ex_ values as a tool in public health interventions and while building recommendation and policy in the field of physical activity. It is postulated that obese children are characterized by an overweight-related decrease in capacity and physical fitness. Consequently, for such children, intense physical exercise is reflected by more rapidly increasing fatigue than observed in slim individuals or those characterized by moderate adiposity. The energy cost of exercise for obese prepubertal children is markedly higher [16]. Similarly, they are characterized by a higher expenditure of energy at rest [20], and their physiologic cost, estimated on the basis of HR, increases markedly more than in slim individuals [25]. Nevertheless, further investigation and analyses are needed to confirm the exclusion of overweight and obese children from the reference values used in screening of cardiorespiratory fitness.

The protocol of the KPR Test is simple. Furthermore, it does not require an examiner with professional qualifications and uses low-cost diagnostic tools. Thus, widespread application of this test in the clinical setting as well as in schools and screening procedures should be considered. However, verification for the usefulness of the KPR Test compared with currently performed objective exercise tests, such as cycloergometry, is required.

As mentioned at the beginning, the KPR Test is one of the step tests used to access cardiorespiratory fitness [9, 10], but it also is used as an endurance test [21]. Further investigation and evaluation comparing the KPR Test with gold standard methods need to be performed with older children who are willing to undergo such testing (i.e., assessment of submaximal VO_2 max_).

## Conclusions

The presented age- and gender-specific reference range for post-exercise HR, determined after the KPR Test and developed on the basis of data from a representative sample of children in Gdansk, enables the assessment and monitoring of submaximal exercise-induced changes in the cardiovascular system and, consequently, the physical fitness of a given individual. Because it is easy to perform and well tolerated by school-aged children (ages 6–12 years), the 3-min step test together with the determination of post-exercise HR (HR_mean post-ex_) can constitute a useful tool for health promoters and educators (physicians, nurses, and teachers). This test makes it possible to estimate the exercise capacity of 6- to 12-year-old children and to monitor changes in the response of the cardiovascular system to submaximal exercise. Consequently, it enables the monitoring of biologic effects associated with physical activity. The presented tables of HR_mean post-ex_ for various age groups of girls and boys can be used for a quick interpretation of results in the setting of a screening study as well as in primary health care. In the authors’ opinion, proper interpretation of the test will enable the development of individualized health programs for children based on measurable values of HR_mean post-ex_, providing proper daily levels of physical activity.
